# Multi-walled carbon nanotubes induce human microvascular endothelial cellular effects in an alveolar-capillary co-culture with small airway epithelial cells

**DOI:** 10.1186/1743-8977-10-35

**Published:** 2013-08-01

**Authors:** Brandi N Snyder-Talkington, Diane Schwegler-Berry, Vincent Castranova, Yong Qian, Nancy L Guo

**Affiliations:** 1 Pathology and Physiology Research Branch, Health Effects Laboratory Division, National Institute for Occupational Safety and Health, 1095 Willowdale Road, Morgantown, WV 26505-2888, USA; 2 Mary Babb Randolph Cancer Center, West Virginia University School of Medicine, Morgantown, WV 26506-9300, USA

**Keywords:** MWCNT, Co-culture, Endothelium, Airway epithelium, Pulmonary inflammation, Pulmonary fibrosis

## Abstract

**Background:**

Nanotechnology, particularly the use of multi-walled carbon nanotubes (MWCNT), is a rapidly growing discipline with implications for advancement in a variety of fields. A major route of exposure to MWCNT during both occupational and environmental contact is inhalation. While many studies showed adverse effects to the vascular endothelium upon MWCNT exposure, *in vitro* results often do not correlate with *in vivo* effects. This study aimed to determine if an alveolar-capillary co-culture model could determine changes in the vascular endothelium after epithelial exposure to MWCNT.

**Methods:**

A co-culture system in which both human small airway epithelial cells and human microvascular endothelial cells were separated by a Transwell membrane so as to resemble an alveolar-capillary interaction was used. Following exposure of the epithelial layer to MWCNT, the effects to the endothelial barrier were determined.

**Results:**

Exposure of the epithelial layer to MWCNT induced multiple changes in the endothelial cell barrier, including an increase in reactive oxygen species, actin rearrangement, loss of VE-cadherin at the cell surface, and an increase in endothelial angiogenic ability. Overall increases in secreted VEGFA, sICAM-1, and sVCAM-1 protein levels, as well as increases in intracellular phospho-NF-κB, phospho-Stat3, and phospho-p38 MAPK, were also noted in HMVEC after epithelial exposure.

**Conclusion:**

The co-culture system identified that alveolar-capillary exposure to MWCNT induced multiple changes to the underlying endothelium, potentially through cell signaling mediators derived from MWCNT-exposed epithelial cells. Therefore, the co-culture system appears to be a relevant *in vitro* method to study the pulmonary toxicity of MWCNT.

## Background

Nanotechnology and the production of nanomaterials for commercial application are fundamental to the innovation of novel technologies in various fields and are projected to become trillion dollar markets in the next decade [1-4]. As the production and use of nanomaterial increases, so will the potential for adverse biological effects due to exposure, particularly by inhalation [1,5]. The unique physico-chemical properties of nanomaterials suggest the potential for unique toxic effects that are mainly attributable to their small size and thus the opportunity for increased uptake and interaction with biological tissues [4]. Multi-walled carbon nanotubes (MWCNT), nanoparticles consisting of concentric layers of cylindrical carbon tubes with a diameter of less than 100 nm, are fibrous nanomaterials known for their light weight, extreme strength and tensile forces, electronic conductivity, and strong capillary forces [6-8]. With a fibrous shape and durability similar to asbestos, exposure to MWCNT is a concern for both short- and long-term lung toxicity [9-11].

*In vivo* aspiration exposure studies of mice to MWCNT reported that MWCNT are biopersistent and induce an acute inflammatory response in the lung followed by a progressive fibrotic state [12,13]. Mice exposed to MWCNT exhibited an increase over control in the number of bronchoalveolar lavage (BAL) polymorphonuclear leukocytes and the activity of lactate dehydrogenase (LDH) in acellular BAL fluid in both a dose- and time-dependent manner [12]. Numerous MWCNT were internalized by and penetrated through the alveolar epithelial cells and alveolar macrophages; furthermore, MWCNT were found to migrate into the interstitium of the alveolar setpa and be transported to the pleural space [13]. Similar results were also obtained in a follow-up experiment involving inhalation of MWCNT [14]. Additionally, in a rat model of MWCNT exposure by intratracheal instillation, increases in LDH levels in BAL fluid, as well as an increase in BAL neutrophil and eosinophil levels, indicated acute inflammation and lung damage [15]. Increased presence of TNF-α and a persistent increase in collagen deposition in the lung indicated that MWCNT induced a fibrotic state [15].

It has been shown that MWCNT exposure induces a broad range of toxic effects both *in vivo* and *in vitro*[16]*.* Production of reactive oxygen species (ROS) was a common effect of MWCNT exposure in multiple cells types, as was the induction of inflammatory markers, such as IL-8, ICAM-1, and MCP-1 [17-19]. MWCNT induced apoptosis [20] and were proposed to induce genotoxic effects by interacting with the mitotic spindle apparatus [21].

Although mono-culture studies of lung epithelial and related cells are the predominant form of *in vitro* nanoparticle toxicological testing, multiple groups have shown a discordant relationship between nanoparticle *in vitro* and *in vivo* effects [22-24]. Current toxicological approaches call for a reduction in observational *in vivo* testing and an increase in predictive *in vitro* analysis, and there is a current push for an increased ability to accurately portray *in vivo* effects in an *in vitro* system [25-27]. As the epithelial lining is the primary barrier to inhaled particles, co-culture of lung epithelial cells, either in submerged or air-liquid interface culture, with associated macrophages, fibroblasts, and/or endothelial cells has increased the potential to study the *in vitro* toxic effects of nanomaterials in a more *in vivo-*like manner [28-36]. Co-culture of multiple cell types has been proposed to be superior to mono-culture exposure and more adequately reflect an *in vivo* signaling environment [31,33,34]. As the respiratory zone has been shown to be the major point of exposure to MWCNT following both aspiration and inhalation of MWCNT *in vivo*[13,14], co-culture of human small airway epithelial cells (SAEC) and human microvascular endothelial cells (HMVEC) was employed to model the alveolar-capillary interaction of the small airways in the lower respiratory tract. Maintenance of the alveolar-capillary unit is essential for efficient pulmonary function [37]. Although inflammation is a necessary response for the repair of lung architecture [38,39], persistent injury to the lung can result in a chronic inflammatory response, loss of alveolar-capillary integrity, and the progressive development of fibrosis [40].

This study employed the co-culture of SAEC, a Type II epithelial cell, and HMVEC to determine the potential toxic effects to the vascular epithelium after epithelial exposure to MWCNT. Through a measure of MWCNT cellular uptake, ROS production, vascular endothelial barrier integrity, angiogenesis, and both extracellular and intracellular cytokine production, this study determined that exposure of the lung epithelial lining to MWCNT may have adverse toxic effects on the underlying endothelium. These adverse effects could be due to signaling from the lung epithelium. The resultant aberrant endothelial activation could result in inflammation and disease states of the lung.

## Results

### Epithelial cells engage MWCNT in co-culture

**Figure 1:**
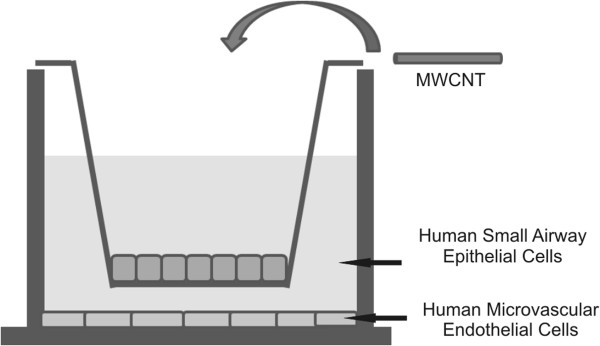
**Co-culture of SAEC and HMVEC.** Human small airway epithelial cells were cultured on the apical side of polyester Transwell inserts (0.4 μm pore size), while human microvascular endothelial cells were cultured in the basolateral chamber of the Transwell. Epithelial cells were exposed through the apical well to MWCNT in DM for various timepoints, and the effects to the underlying endothelium were assayed.

**Figure 2:**
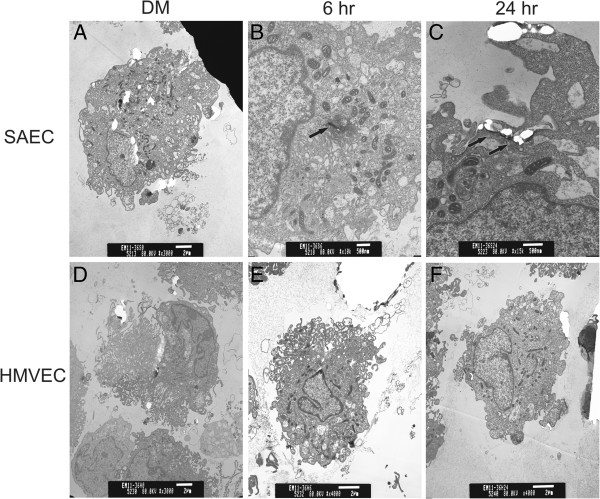
**TEM of SAEC and HMVEC following addition of MWCNT to the apical Transwell chamber.** SAEC and HMEC were grown in co-culture, followed by exposure of SAEC to DM or 1.2 μg/ml MWCNT for 6 or 24 h. Cells were washed thoroughly and collected for TEM. MWCNT were found within SAEC (arrows) but not in HMVEC from the basolateraly Transwell chamber.

Previous studies showed that both SAEC and HMVEC in mono-culture engaged MWCNT at the cell periphery as well as engulfed the nanotubes [19,41]. To determine if MWCNT had the ability to interact with epithelial or endothelial cells in co-culture, SAEC and HMVEC were grown in co-culture, with SAEC on the Transwell membrane and HMVEC in the basolateral chamber as described in the Materials and Methods (Figure 1), and SAEC exposed to either dispersion media (DM) or 1.2 μg/ml MWCNT for 6 or 24 h. Following exposure, SAEC and HMVEC were collected and analyzed by transmission electron microscopy (TEM) for the presence of MWCNT. At both 6 and 24 h, SAEC interacted with MWCNT (Figure 2A-C),while MWCNT were not apparent in the endothelial cell preparations (Figure 2D-F). MWCNT had the ability to interact with the epithelial barrier but did not appear to pass through the Transwell membrane to the endothelial layer. Therefore, it was hypothesized that effects to the endothelium surveyed after epithelial exposure are primarily due to MWCNT interaction with and any concurrent downstream signals from the epithelial cells and not due to direct contact of the endothelial cells with MWCNT. Although the possibility of endothelial interaction with low amounts of MWCNT that may enter the basolateral chamber cannot be ruled out, this does not appear to be the major route of endothelial activation.

### Epithelial exposure to MWCNT induced the secretion of inflammatory mediators in co-culture

**Figure 3:**
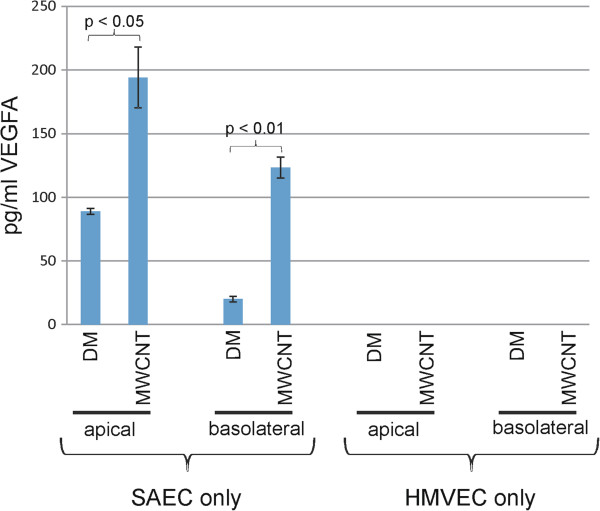
**Cell mediators from SAEC exposure can pass through the Transwell membrane.** SAEC were grown in the apical Transwell chamber without the presence of HMVEC in the basolateral well. HMVEC were grown in the basolaeral well without the presence of SAEC in the apical chamber. The apical chamber was exposed to DM or 1.2 μg/ml MWCNT for 24 h. Apical and basolateral media were collected for analysis by VEGFA ELISA. All values given are the mean ± standard error. SAEC only VEGFA levels increased from 89.04 ± 2.27 pg/ml to 194.04 ± 23.85 pg/ml in the apical well and 20.00 ± 2.06 pg/ml to 123.33 ± 8.13 pg/ml in the basolateral chamber. HMVEC only VEGFA levels were not above the level of detection of the ELISA assay.

MWCNT were noted *in vivo* to invoke an acute inflammatory response in the lung after aspiration exposure followed by a persistent fibrotic response [12]. To determine whether cellular mediators released following SAEC exposure could pass through the Transwell membrane to the underlying HMVEC, SAEC were cultured in the apical Transwell chamber without HMVEC in the basolateral chamber (SAEC alone). Conversely, HMVEC were cultured in the basolateral chamber without SAEC in the apical chamber (HMVEC alone). Each culture system was exposed to either DM or 1.2 μg/ml MWCNT in the apical well for 24 h. Following exposure, media was removed from both the apical and basolateral chambers. Expression levels of vascular endothelial growth factor A (VEGFA) in the apical and basolateral chambers following MWCNT exposure were analyzed by an enzyme-linked immunosorbent assay (ELISA) (Figure 3). In SAEC alone cultures, VEGFA levels increased from 89.04 ± 2.27 pg/ml to 194.04 ± 23.85 pg/ml in the apical chamber after MWCNT exposure, while protein levels increased from 20.00 ± 2.06 pg/ml to 123.33 ± 8.13 pg/ml in the basolateral chamber. Neither DM nor MWCNT exposure to the apical chamber of the culture consisting of HMVEC alone in the basolateral chamber gave appreciable VEGFA levels above the threshold of detection of the ELISA assay. As VEGFA protein levels increased in the basolateral chamber after SAEC exposure, it was concluded that cellular mediators secreted by SAEC were capable of passing through the Transwell membrane to the basolateral chamber, thus potentially affecting the underlying endothelial layer.

**Figure 4:**
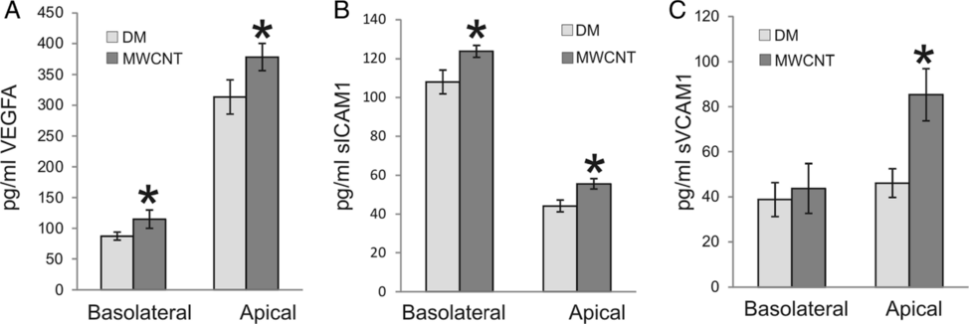
**SAEC exposure to MWCNT increases the expression of secreted inflammatory mediators in coculture.** SAEC and HMVEC were grown in the apical and basolateral chambers, respectively, of a co-culture system and SAEC exposed to 1.2 μg/ml MWCNT for 24 hours. Apical and basolateral media were collected and assayed by ELISA for VEGFA, sICAM1, and sVCAM1 protein expression. All values given are the mean ± standard error. VEGFA levels **(A)** increased from 87.15 ± 6.58 pg/ml to 114.96 ± 14.89 pg/ml in the basolateral chamber and 313.45 ± 27.85 pg/ml to 378.38 ± 21.89 pg/ml in the apical chamber. sICAM1 levels **(B)** increased from 107.99 ± 6.14 pg/ml to 123.76 ± 3.00 pg/ml in the basolateral chamber and 44.13 ± 3.04 pg/ml to 55.56 ± 2.63 pg/ml in the apical chamber. sVCAM1 levels **(C)** increased from 38.77 ± 7.52 pg/ml to 46.07 ± 11.07 pg/ml in the basolateral chamber and 43.69 ± 6.38 pg/ml to 85.29 ± 11.64 pg/ml in the apical chamber. * p < 0.05 above control.

To determine the levels of inflammatory mediators in co-culture, VEGFA and two other secreted proteins known to have a role in the inflammatory process, soluble intracellular adhesion molecular 1 (sICAM-1) and soluble vascular cell adhesion molecule 1 (sVCAM-1), were assayed for their protein levels in co-culture media after epithelial exposure. SAEC and HMVEC were grown in co-culture for 72 h, serum-starved overnight, and SAEC exposed to DM or 1.2 μg/ml for 24 h. Following exposure, media were removed from both the apical and basolateral chambers of the co-culture and assayed for these inflammatory protein markers by ELISA. Protein levels of VEGFA increased significantly from 87.15 ± 6.58 pg/ml in the basolateral chamber to 114.96 ± 14.89 pg/ml after MWCNT exposure (Figure 4A). VEGFA protein levels also increased significantly from 313.45 ± 27.85 pg/ml in the apical chamber to 378.38 ± 21.89 pg/ml after MWCNT exposure (Figure 4A). Significant increases in sICAM-1 and sVCAM-1 were also noted. sICAM-1 protein levels increased significantly in the apical well from 44.13 ± 3.04 pg/ml to 55.56 ± 2.63 pg/ml, as well as in the basolateral chamber from 107.99 ± 6.14 pg/ml to 123.76 ± 3.00 pg/ml, following MWCNT exposure (Figure 4B). sVCAM-1 protein levels increased significantly in the apical well from 43.69 ± 6.38 pg/ml to 85.29 ± 11.64 pg/ml after epithelial exposure to MWCNT (Figure 4C). There was no significant increase in sVCAM-1 levels in the basolateral chamber after exposure with an increase of 38.77 ± 7.52 pg/ml to 46.07 ± 11.07 pg/ml.

### Epithelial exposure to MWCNT induced superoxide radical production in HMVEC and disrupted the endothelial barrier

**Figure 5:**
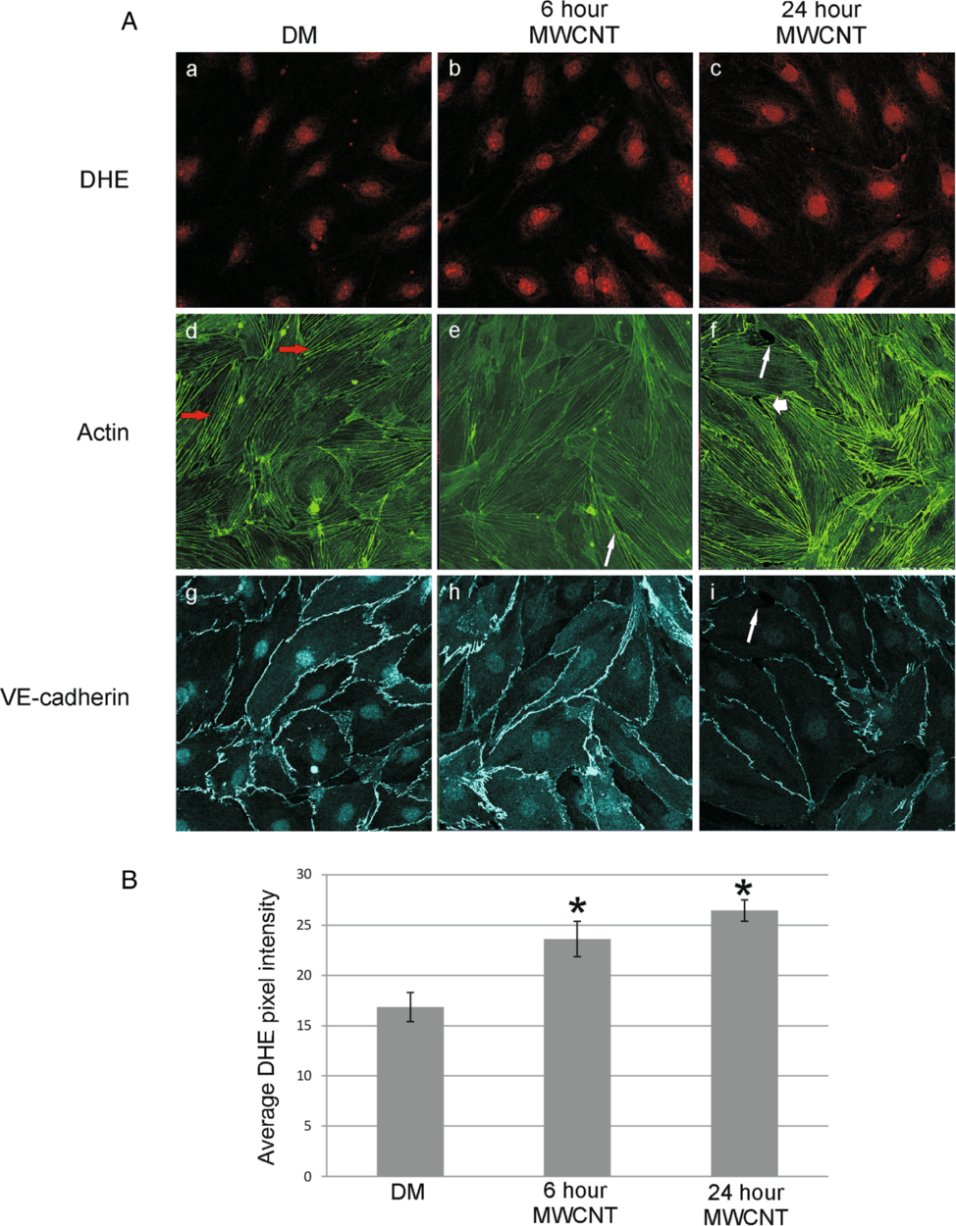
**SAEC exposure to MWCNT induces ROS production and morphological changes in HMVEC. A**. SAEC and HMVEC were grown in the apical and basolateral chamber, respectively, of a co-culture system and SAEC exposed to 1.2 μg/ml MWCNT for 6 or 24 h. ROS production **(a**-**c)** was assayed by the addition of 5 mM dihydroethidium to the basolateral media 30 min prior to fixation for confocal microscopy. Actin filaments **(d**-**f)** and VE-cadherin **(g**-**i)** were visualized by antibody staining. Red arrows show stress fibers, long white arrows show cellular gaps, and short white arrows show areas of actin ruffling. **B**. DHE fluorescence pixel intensity was quantified on the acquired confocal images using Optimas 6.51 software. A minimum of 10 cells were scored for each sample from 4 independent experiments. Data are presented as mean ± SE. There was a significant increase in average DHE pixel intensity at both 6 (23.62 ± 1.75) and 24 h (26.47 ± 1.06) over DM control cells (16.84 ± 1.45). * p < 0.05.

MWCNT have been shown to increase ROS levels in both SAEC and HMVEC in mono-culture following MWCNT exposure [19,41]. To determine if SAEC exposed to MWCNT had the ability to increase ROS production in HMVEC, SAEC and HMVEC were grown in co-culture and SAEC in the apical chamber exposed to either DM or 1.2 μg/ml MWCNT for 6 or 24 h. Dihydroethidium (DHE) exhibits nuclear localization and a red fluorescence upon oxidation by superoxide radical (O_2_^-^), a major form of ROS, and was added at a final concentration of 5 μM to the basolateral chamber for the final 30 minutes of exposure. Both control and experimental HMVEC were simultanesouly fixed and stained for immunofluorescence, and, under uniform confocal microscopy parameters, an increase in O_2_^-^ production by HMVEC in the basolateral chamber was seen upon increasing time of exposure of SAEC in the apical chamber to MWCNT (Figure 5A, a-c). The pixel intensity of the DHE staining in 4 independent experiments was quantified using Optimas 6.51 software and is presented as the mean ± standard error (Figure 5B). There was a significant increase (p < 0.05) in average DHE pixel intensity at both 6 (23.62 ± 1.75) and 24 h (26.47 ± 1.06) over DM control cells (16.84 ± 1.45).

A disruption of endothelial barrier integrity was noted in HMVEC after SAEC exposure. The pulmonary endothelium acts as a semi-permeable barrier, and integrity of the endothelial barrier is necessary for efficient pulmonary function [37]. The HMVEC cytoskeleton displayed altered actin arrangement, with increasing time of SAEC exposure, resulting in membrane ruffling, loss of stress fiber formation, and gap formation between HMVEC cells (Figure 5d-f). This gap formation and loss of cell-cell contacts was concurrent with a loss of VE-cadherin at the cell periphery (Figure 5g-i). VE-cadherin expression in the adherens junctions of endothelial cells is necessary for the maintenance of low vascular permeability, and this process was potentially disrupted in HMVEC after SAEC exposure.

### Epithelial exposure to MWCNT increased the angiogenic ability of endothelial cells

**Figure 6:**
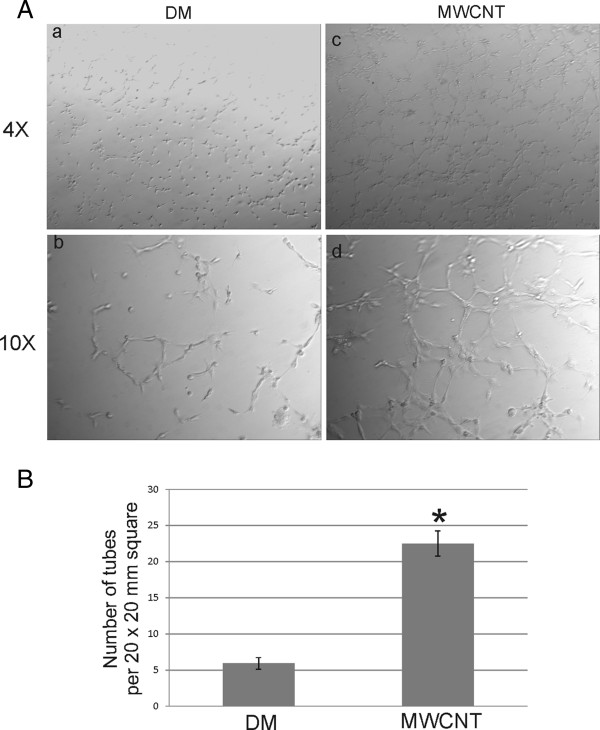
**SAEC exposure to MWCNT increases the angiogenic potential of HMVEC. A**. SAEC and HMVEC were grown in the apical and basolateral chambers, respectively, of a co-culture system, and SAEC were exposed to either DM or 1.2 μg/ml MWCNT for 24 h. HMVEC were removed from the co-culture, rinsed thoroughly in serum free media, and plated in serum free media on Matrigel plugs. Images of capillary-like formation were captured 4 h after plating at both 4X and 10X magnifications in DM **(a**,**b)** and MWCNT **(c**,**d)** exposures. Three separate angiogenesis assays were performed, and a representative image is shown. **B**. The number of tube-forming cells in DM or MWCNT-exposed co-cultures was determined by counting the number of tube-forming cells in 6 randomly chosen 20 × 20 mm squares in the 4X images of three separate angiogenesis assays. The average number ± standard error of tube-forming cells in the DM control was 5.91 ± 0.81, and the mean ± standard error of tube-forming cells in the MWCNT-exposed co-culture was 22.50 ± 1.73. * p < 0.05.

A role of endothelial cells is to reform the vasculature and, upon injury, respond through physiological angiogenesis to form new blood vessels in a restricted manner [42]. Pathological angiogenesis is a known hallmark of a variety of diseases, such as rheumatoid arthritis and cancer, and is necessary for the progression of pulmonary diseases, such as fibrosis [39,42]. To determine if epithelial exposure to MWCNT increased the angiogenic potential of the endothelium, SAEC and HMVEC were grown in the apical and basolateral chambers, respectively, of a co-culture system, and SAEC were exposed to either DM or 1.2 μg/ml MWCNT for 24 h. Following exposure, HMVEC were removed from the co-culture system, rinsed with serum free media, and placed into serum free media on a Matrigel plug where they were allowed to form capillary-like structures for 4 h. HMVEC cells from DM exposed co-cultures had minimal tube formation and the majority remained as single cells 4 hours after plating (Figure 6A, a-b). Conversely, HMVEC from MWCNT exposed co-cultures had extensive capillary-like structure formation (Figure 6A, c-d). To determine the average number of tubes formed in both DM and MWCNT exposed co-cultures, 6 20 × 20 mm squares were randomly chosen in the 4X images of 3 independent angiogenesis assays and the number of tubes counted. There was a significant increase (p < 0.05) in the average number of tubes in the MWCNT-exposed co-culture (22.50 ± 1.73) than in the DM exposed control (5.91 ± 0.81) (Figure 6B). As HMVEC had been removed from the co-culture system and rinsed free of co-culture cellular mediators, this enhanced angiogenic ability was possibly due to cellular signaling from SAEC after MWCNT exposure affecting innate changes in the endothelial barrier.

### Epithelial exposure to MWCNT induced the expression of intracellular inflammatory signals in HMVEC

**Figure 7:**
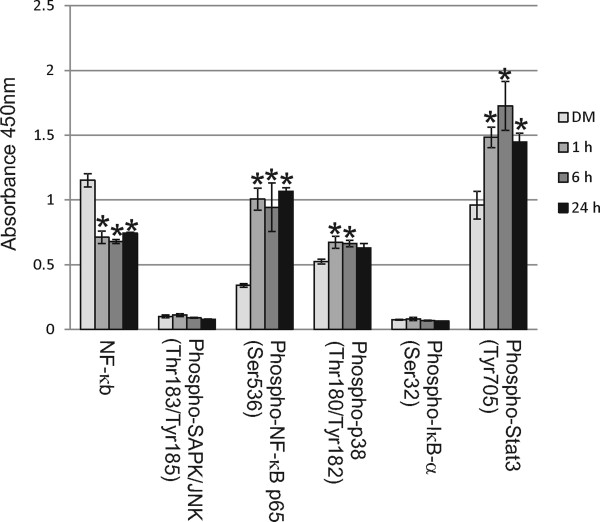
**SAEC exposure to MWCNT increases the expression of cellular inflammatory signals in HMVEC.** Two biological replicates of SAEC and HMVEC were grown in the apical and basolateral chambers, respectively, of a co-culture system, and SAEC was exposed to DM or 1.2 μg/ml MWCNT for 1, 6 or 24 h. HMVEC were lysed and protein levels of intracellular inflammatory signals read at an absorbance of 450 nm using a PathScan Inflammation Multi-Target Sandwich ELISA from Cell Signaling Technology. All values given are the mean ± standard error. NF-κB had a significant decrease in absorbance over time (DM, 1.152 ± 0.051; 1 h, 0.713 ± 0.048; 6 h, 0.6795 ± 0.0145; 24 h, 0.7445 ± 0.0055). Phospho-NF-κB (Ser536) (DM, 0.341 ± 0.013; 1 h, 1.0065 ± 0.0855; 6 h, 0.944 ± 0.187; 24 h, 1.068 ± 0.026) and phospho-Stat3 (Tyr705) (DM, 0.96 ± 0.107; 1 h, 1.483 ± 0.079; 6 h, 1.7265 ± 0.1895; 24 h, 1.446 ± 0.07) had significant increases in absorbance over time, while phospho-p38 (Thr180/Tyr182) (DM, 0.5245 ± 0.0185; 1 h, 0.673 ± 0.047; 6 h, 0.6645 ± 0.0255; 24 h, 0.6285 ± 0.0345) had a significant increase at 1 and 6 h.* p < 0.05. Phospho-SAPK/JNK (Thr183/Tyr185) (DM, 0.101 ± 0.011; 1 h, 0.112 ± 0.011; 6 h, 0.0905 ± 0.0025; 24 h, 0.08 ± 0.002) and phospho-IκB-α (Ser32) (DM, 0.0765 ± 0.0025; 1 h, 0.082 ± 0.013; 6 h, 0.0695 ± 0.0035; 24 h, 0.066 ± 0.002) did not have a significant increases in absorbance.

In addition to secreted inflammatory factors, a number of intracellular inflammatory proteins were altered in HMVEC after SAEC exposure to MWCNT. SAEC and HMVEC were grown in the apical and basolateral chambers, respectively, of a co-culture system, serum-starved overnight, and SAEC exposed to DM or 1.2 μg/ml for 24 h. Following exposure, HMVEC were lysed and cell lysates assayed at absorbance 450 nm for protein levels of NF-κB, phospho-SAPK/JNK, phospho-NF-κB p65, phospho-p38, phospho-IκB-α, and phospho-Stat3 using a Pathscan Inflammation Multi-Target Sandwich ELISA from Cell Signaling Technology. Although a significant decrease (p < 0.05) was seen in overall NF-κB levels in HMVEC after SAEC exposure to MWCNT (DM, 1.152 ± 0.051; 1 h, 0.713 ± 0.048; 6 h, 0.6795 ± 0.0145; 24 h, 0.7445 ± 0.0055), there was a significant increase (p < 0.05) in phospho-NF-κB p65 (Ser536) (DM, 0.341 ± 0.013; 1 h, 1.0065 ± 0.0855; 6 h, 0.944 ± 0.187; 24 h, 1.068 ± 0.026) and phospho-Stat3 (Tyr705) (DM, 0.96 ± 0.107; 1 h, 1.483 ± 0.079; 6 h, 1.7265 ± 0.1895; 24 h, 1.446 ± 0.07) at 1, 6 and 24 h post-exposure (Figure 7). A significant increase (p < 0.05) in phospho-p38 MAPK (Thr180/Tyr182) (DM, 0.5245 ± 0.0185; 1 h, 0.673 ± 0.047; 6 h, 0.6645 ± 0.0255; 24 h, 0.6285 ± 0.0345) was also noted at 1 and 6 hrs post-exposure (Figure 7a). No significant change (p > 0.05) was seen in the levels of phospho-SAPK/JNK (Thr183/Tyr185) (DM, 0.101 ± 0.011; 1 h, 0.112 ± 0.011; 6 h, 0.0905 ± 0.0025; 24 h, 0.08 ± 0.002) or phospho-IκB-α (Ser32) (DM, 0.0765 ± 0.0025; 1 h, 0.082 ± 0.013; 6 h, 0.0695 ± 0.0035; 24 h, 0.066 ± 0.002) in HMVEC after SAEC exposure to MWCNT (Figure 7).

## Discussion

Mono-culture of cells is the predominant form of cell culture for toxicological testing; however, mono-culture results are not always concordant with results obtained *in vivo*[22-24]. Co-culture is a promising alternative to mono-culture and provides a more *in vivo*-like environment for the determination of toxicological results [16,28-30,32,34]. Cross-talk between different cell types in co-culture may elicit cellular reactions that are different from those which occur in mono-culture. Co-culture of SAEC and HMVEC models the alveolar-capillary interaction, which is a critical barrier against inhaled particles such as MWCNT. As MWCNT have been shown to enter the lung epithelial cells *in vivo*[13], the aim of this study was to determine if the interaction of lung epithelial cells with MWCNT *in vitro* could affect the underlying microvascular. This aim was examined through the use of a relevant alveolar-capillary co-culture model so as to allow cell-cell communication between the epithelial and endothelial cells to analyze the microvasvular effects in a manner more akin to *in vivo* signaling.

MWCNT were shown previously to enter the lung epithelium *in vivo* and also be taken up by SAEC *in vitro*[13,14,41]. *In vivo* exposure to MWCNT resulted in the majority of MWCNT depositing in the alveolar region following both aspiration and inhalation *in vivo* exposure, with penetration of MWCNT across the lung epithelium into the alveolar interstitium, and progressive development of fibrosis [13,14]. Exposure of SAEC to MWCNT resulted in an increase in ROS production, protein phosphorylation, cellular migration, and the release of inflammatory cytokines [41]. Co-culture of SAEC with HMVEC did not appear to alter the ability of SAEC to engulf MWCNT. As SAEC were previously shown to elicit a number of cellular effects upon MWCNT exposure [41], this study determined that cellular mediators secreted by SAEC could affect surrounding cells. No MWCNT were detected in the HMVEC preparations; therefore, it was determined that direct MWCNT exposure was not the primary source of cellular changes in HMVEC.

ROS, the collective term for the intermediates formed during oxidative metabolism that includes O_2_^-^, hydrogen peroxide (H_2_O_2_), hydroxyl radical (·OH), and peroxynitrite (ONO_2_^-^), function as both antimicrobial and key signaling molecules [43] and are involved in various signaling pathways. Aberrant ROS production can result in indiscriminate damage to DNA, proteins, and lipids in cells [44]. Particularly in the pulmonary system, an overproduction of ROS is correlated with endothelial dysfunction and pulmonary hypertension [45]. The increase of O_2_^-^ production in HMVEC indicates that an alteration in ROS signaling in HMVEC may be seen after SAEC exposure to MWCNT.

HMVEC displayed membrane ruffling, actin reorganization, and gap formation between the endothelial cells of the monolayer with increasing time of SAEC exposure to MWCNT. This phenomenon had been previously shown to occur in HMVEC following the direct exposure of endothelial cells to MWCNT and resulted in an increase in vascular permeability [19]. Preservation of pulmonary endothelial monolayer integrity is necessary for efficient pulmonary function, and disruption may allow for the transport of fluid and molecular activators into the alveolar region, thus resulting in decreased gas exchange and inflammation [37]. *In vivo* exposure of rats suggested endothelial dysfuction following inhalation of MWCNT [46]. Integral to the integrity of the endothelial monolayer is the cytoskeleton of the endothelial cells. The maintenance of proper vascular tone is essential to prevent vascular failure [47]. Loss of actin organization in HMVEC suggested dysfunction in the endothelial monolayer and suggests a potential critical pulmonary situation in the lung following epithelial exposure to MWCNT. Also required for the integrity of the vascular endothelial monolayer is the proper functioning of the endothelial junctions [48]. Junctional contacts between endothelial cells act as semi-permeable barriers to allow the regulated passage of molecules between the vascular and interstitial spaces [49]. One molecule that plays a key role in the maintenance of these junctions is VE-cadherin. VE-cadherin is restricted to endothelial cells and is a major component of intercellular junctions [50]. Proper VE-cadherin function is necessary for development, and loss of VE-cadherin results in an increase in vascular permeability, thus permitting passage of fluid and macromolecules across the vascular barrier and resulting in inflammation and edema [51]. The loss of the VE-cadherin signal at the cell surface of HMVEC after SAEC exposure to MWCNT, coupled with cell membrane ruffling and gap formation, suggests that the endothelial barrier had been compromised.

Exposure of SAEC to MWCNT also increased the angiogenic ability of the HMVEC cells. Physiological angiogenesis is a common occurrence in processes such as wound healing, which are self-limited in time, but pathological angiogenesis may occur unregulated for longer time periods [42]. Chronic inflammatory diseases depend upon chronic neovascularization, and MWCNT have been shown to induce chronic inflammation with a shift to fibrosis in MWCNT-exposed mice [12,14,42]. Exposure of SAEC to MWCNT upregulates the angiogenic potential of HMVEC, suggesting that MWCNT exposure can induce the wound healing process and may potentially induce pathological angiogenesis. As no MWCNT were found to pass through the Transwell system into the endothelial well, cellular mediators secreted by the epithelial cells following MWCNT exposure appear to be the factor behind these endothelial cellular effects.

One of the main regulators of angiogenesis is VEGFA. VEGFA in the lung is released mainly by the epithelium, has a high specificity for endothelial cells, and is crucial for the maintenance of lung structure [52]. VEGFA expression plays a role in endothelial cell survival, cell proliferation and migration, vasodilation, and enhanced vessel permeability [52]. Expression of VEGFA is known to have a pathological role when dysregulated, and its increased expression plays a role in the development of inflammation, edema, and cancer [53]. One mechanism behind increased endothelial cell permeability is VEGFA-induced endocytosis of VE-cadherin. As loss of VE-cadherin-mediated adhesion results in increased vascular permeability, VEGFA has the ability to induce an increase in vascular permeability through the loss of VE-cadherin signaling [54]. The increase in angiogenic ability of HMVEC coupled with loss of VE-cadherin expression at the cell surface suggests that VEGFA may be playing a role in MWCNT-induced cellular signaling. Indeed, soluble VEGFA protein levels were shown to increase in the Transwell system following SAEC exposure to MWCNT, and this increase in expression correlated with increased angiogenesis and enhanced vascular permeability in HMVEC.

In addition to VEGFA, we chose to determine the levels of soluble intercellular adhesion markers, sICAM1 and sVCAM1, to determine their expression following SAEC exposure to MWCNT. ICAM1 and VCAM1, and their soluble forms, support the interaction of leukocytes with endothelial cells and are necessary for their transmigration [55]. sICAM-1 and sVCAM-1 play a role in the development and severity of various diseases, such as cardiovascular disease, cancer, and autoimmune diseases [56,57]. Both sICAM-1 and sVCAM-1 have been suggested to play a role in the upregulation of intracellular inflammatory mediators and endothelial dysfuction, resulting in endothelial cell migration, tube formation, and increased angiogenesis [56]. Detection of sICAM-1 and sVCAM-1 in patients may provide a marker for the severity of disease and potential disease outcome. As the soluble cellular mediators in our system have the ability to pass back and forth through the Transwell membrane, it is not possible at this time to determine from which cell type the mediator originated. However, an overall increase of these soluble mediators is seen in the co-culture system, suggesting that cell-cell communication can occur between the epithelial and endothelial cells. Intracellular inflammatory signals involved in key regulatory pathways of inflammation were also assayed in HMVEC to determine innate changes within the cells. Phosphorylation of NF-κB and STAT3 is known to induce gene activation in various processes, such as proliferation, transformation, and apoptosis [58,59]. NF-κB has been suggested to be a master regulator of inflammation, and its role is essential in the coordinated response to lung injury [59]. NF-κB activation in endothelial cells has been shown to increase vascular permeability as well as upregulate the expression of cellular adhesion molecules, such as ICAM1 and VCAM1 [59]. The activation of STAT3 in HMVEC following SAEC exposure to MWCNTs suggests a reaction to an induced inflammatory response and maintenance of cellular homeostasis [58]. Sustained activation of STAT3 has been suggested to play a critical role in the pathogenesis of lung fibrosis by differentially inducing both apoptotic and proliferative cellular signals [60]. Phosphorylation of the serine/threonine mitogen-activated protein kinase p38 occurs in response to inflammatory signals and results in the activation of proinflammatory mediators [61]. p38 activation has been shown to regulate the expression of ICAM1 and VCAM1 [62] and induce cytoskeletal remodeling in endothelial cells [63]. Upregulated phosphorylation of the HMVEC intracellular inflammatory signals NF-κB, STAT3, and p38 MAPK in the co-culture system following SAEC exposure to MWCNT suggests that cellular mediators from the epithelial cells following exposure are able to elicit a response from the endothelial cells, which in turn respond by expression of their own cellular signaling. This cross-talk between the two cell types is a benefit of co-culture over mono-culture in that cellular signaling pathways can be relayed back and forth between the cell types to elicit a response which is more realistic to the *in vivo* environment.

The proper evaluation of current and newly created nanomaterials requires an efficient and relevant model system. As mono-culture studies of nanomaterial exposure have resulted in discordant results from those seen during *in vivo* exposure, we chose to develop a co-culture system in which to test the toxicity of epithelial exposure to MWCNT on the vascular endothelium in a relevant manner. A correlation study of global mRNA and miRNA expression between MWCNT exposure in SAEC and HMVEC mono-culture separately, SAEC and HMVEC in co-culture, and mouse lungs exposed to MWCNT determined that changes in gene expression due to MWCNT exposure in co-culture were highly correlated with those seen *in vivo*, while mono-culture exposure did not correlate well with the *in vivo* results (Snyder-Talkington, in preparation). Therefore, our co-culture system was a relevant model in which to test the potential lung toxicity of MWCNT to the vascular endothelium. Using this co-culture model, we determined that epithelial exposure to MWCNT induced multiple changes to the underlying endothelium, potentially through cell signaling mediators derived from MWCNT-exposed epithelial cells.

## Conclusions

In conclusion, this study determined that co-culture of SAEC and HMVEC could be used to elucidate the toxic effects to the vascular epithelium after epithelial exposure to MWCNT. Through the measure of MWCNT cellular uptake, ROS production, vascular endothelial barrier integrity, angiogenesis, and both extracellular and intracellular mediator production, this study determined that exposure of the lung epithelial lining to MWCNT may have adverse toxic effects on the underlying endothelium. These adverse effects could be due to cell signaling mediators from lung epithelial cells, which result in endothelial cell dysfunction and potential inflammation and disease states of the lung. The co-culture model was a relevant alveolar-capillary model to study the implications of inhalation exposure to MWCNT and may help to elucidate the signaling pathways induced by and potential hallmarks of exposure to MWCNT.

## Materials and methods

### MWCNT

MWCNT used in the present study were a gift from Mitsui-&-Company (MWCNT-7, lot # 05072001 K28). Characterization of MWCNT has been previously described [12]. Briefly, the bulk MWCNT exhibited a distinctive crystalline structure with the number of walls ranging from 20 to 50 walls. Overall, MWCNT trace metal contamination was 0.78%, including sodium (0.41%) and iron (0.32%) with no other metals present above 0.02%. The quantitative analysis of TEM micrographs revealed that the median length of this MWCNT sample was 3.86 μm (GSD 1.94) and the count mean width was 49 ±13.4 (S.D.) nm. The zeta potential of the MWCNT in DM was determined to be -11 mV.

### MWCNT preparation

For cell culture studies, MWCNT were prepared in dispersion medium [64]. TEM micrographs of MWCNT dispersed in DM demonstrated that DM promotes significant dispersion of MWCNT. Briefly, DM consisted of phosphate-buffered saline (PBS), pH 7.4, Ca/Mg free supplemented with 5.5 mM D glucose, 0.6 mg/ml serum albumin, and 0.01 mg/ml 1,2 dipalmitoyl-sn-glycero-3-phosphocholine (DPPC, Sigma). DPPC was prepared fresh as a 1 mg/ml stock solution in absolute ethanol. MWCNT were prepared in DM followed by indirect sonication at 4°C for 5 min (Hielscher ultrasonic processor, UIS259L) at amplitutide 100% and cycle 1. After the indirect sonication, the suspension was directly sonicated for 5 min at 5 W output and 10% duty cycle (Branson Sonifier 450). The stock solution (0.5 mg/ml) of MWCNT was kept at 4°C and used within 2–3 weeks. Prior to cell culture experiments, the MWCNT stock solution was directly sonicated for 1 min at the setting indicated above.

MWCNT were used at a concentration of 1.2 μg/ml in all experiments. Based upon *in vivo* alveolar surface area, occupationally observed MWCNT airborne concentrations, MWCNT mass median aerodynamic diameter, and minute ventilation, a 1.2 μg/ml concentration of MWCNT was extrapolated to correspond to previously identified *in vivo* exposures of MWCNT which induced transient and chronic cellular signaling [12,65,66].

### Cell culture

SAEC were a kind gift from Dr. Tom K. Hei (Columbia University, New York, NY) [67]. SAEC were cultured in serum free complete SAGM medium supplemented with various growth factors supplied by the manufacturer (Lonza Walkersville, Inc., Walkersville, MD). HMVEC were a kind gift from Dr. Rong Shao (Biomedical Research Institute, Baystate Medical Center/University of Massachusetts, Amherst, Springfield, MA) and were cultured as previously described [68]. Briefly, HMVEC were grown in endothelial basal medium-2 (EBM-2) (Lonza) and supplemented with 10% fetal bovine serum (FBS, Atlanta Biological, Lawrenceville, GA), 100 U/ml penicillin and 10 μg/ml streptomycin (Lonza), 0.01 μg/ml epidermal growth factor (EGF, Sigma), and 1 μg/ml hydrocortisone (Sigma). All cells were maintained in an incubator at 37°C with 5% CO_2_.

To prepare co-cultures, inserts were removed from a 6-well polyester Transwell with a 0.4 μm pore size (Corning, Tewksbury, MA) and hydrated in SAEC complete media in a companion 6-well dish for at least 1 hour. HMVEC were plated at 200,000 cells per well at the bottom of each well of the Transwell with or without coverslips and allowed to adhere for at least 1 h without apical chamber inserts. Inserts were returned to the Transwell after hydration, and 150,000 SAEC were plated onto the Transwell insert. Cells were maintained in 2.5 ml complete EBM-2 media in the basolateral chamber and 1.5 ml complete SAEC media in the apical chamber. Cells were allowed to form intact epithelial and endothelial barriers for 72 h before serum starvation and exposure to MWCNT(1.2 μg/ml ≈ 0.33 μg/cm^2^cells) (Figure 1).

### Transmission electron microscopy

SAEC and HMVEC interaction with and uptake of MWCNT were analyzed by TEM. SAEC and HMVEC were plated at 150,000 and 200,000 cells, respectively, per Transwell, allowed to form intact epithelial and endothelial barriers over 72 h, and serum starved. SAEC were exposed to MWCNT (1.2 μg/ml) for 6 or 24 h. After exposure, cells were washed with ice cold Ca^2+^/Mg^2+^ free PBS, scraped from their wells, and harvested by centrifugation at 109 × g for 5 min. Cells were fixed in Karnovksy’s fixative (2.5% glutaraldehyde and 3% paraformaldehyde in 0.1 M sodium cacodylate, pH 7.4), washed 3 times in 0.1 M sodium cacodylate, and postfixed in 1% osmium tetraoxide followed by washing with 0.1 M sodium cacodylate and distilled water. The cells were dehydrated by sequential washings in 25, 50, and 100% ethanol and then embedded in LX-112 (Ladd, Williston, VT). The ultrathin sections were stained with uranyl acetate and lead citrate and examined with a transmission electron microscope (JEOL 1220, Tokyo).

### ELISA

Three independent biological replicates of SAEC and HMVEC were plated at 150,000 and 200,000 cells, respectively, per well of a 6-well polyester Transwell and grown at 37°C for 72 h. Cells were serum starved overnight followed by epithelial exposure to either DM or 1.2 μg/ml MWCNT for 6 or 24 h. Conditioned media from each biological replicate was collected and assayed in triplicate for VEGF, sVCAM-1, and sICAM-1 protein expression levels using DuoSet ELISA Development Systems from R&D Systems according to manufacturer’s protocol. Statistical analysis was conducted using a two-sample *t*-test assuming unequal variances.

### Confocal microscopy

ROS measurements by confocal microscopy were performed according to the methods previously described [69]. Briefly, SAEC were cultured in on the Transwell membrane and HMVEC cultured on coverslips in the basolateral chamber for 72 h. Cells were serum starved overnight, and SAEC were exposed to either DM or 1.2 μg/ml MWCNT. DM and 24 h exposures were exposed to DM or MWCNT after serum starvation. Six hours prior to the end of the 24 h exposure period, the 6 h MWCNT exposure was begun to ensure that all cells were grown in co-culture for the same amount of time. During the exposure periods, DHE (Invitrogen) was added to the basolateral chamber at a final concentration of 5 μM for the last 30 min of exposure. After incubation, HMVEC were fixed with 4% paraformaldehyde and permeabilized with 0.1% Triton X-100/PBS. HMVEC were incubated with anti-actin (AlexaFluor 546, Invitrogen) and rabbit anti-VE-cadherin (Sigma), washed three times with PBS, incubated with anti-rabbit AlexaFluor 647 (Invitrogen), and mounted on slides with ProLong Gold anti-fade (Invitrogen). A Zeiss LSM 510 microscope was used to obtain images, and a representative image of each exposure time was obtained. Scale bars were generated and inserted by LSM Zen 2011 Light Edition software. Three indepdent biological replicates were analyzed, and a representative image is shown.

### Angiogenesis assay

Three independent biological replicates of SAEC and HMVEC were plated at 150,000 and 200,000 cells, respectively, per well of a 6 well polyester Transwell and grown at 37°C for 72 h. SAEC and HMVEC were serum starved overnight, and SAEC were exposed to either DM or 1.2 μg/ml MWCNT for 24 h. Following exposure, HMVEC were trypsinized from the well and washed two times with PBS. Three technical replicates of each biological replicate were plated. 30,000 HMVEC were placed into 150 μl of Matrigel (BD Biosciences) in a 24-well dish and allowed to grow for 4 hours. Angiogenesis was imaged on an Olympus IX70 with a Retiga 2000R camera (QImaging, Surrey, British Columbia, Canada). Six randomly chosen 20 × 20 mm squares were chosen in the 4X images of 3 separate angiogenesis assays to determine the average number of tube-forming cells in each square.

### PathScan inflammation multi-target sandwich ELISA

Two independent biological replicates of SAEC and HMVEC were plated at 1.5 million and 2 million cells, respectively, per plate of 100 mm polycarbonate Transwell dishes and grown at 37°C for 72 h. Cells were serum starved overnight, followed by epithelial exposure to either DM or 1.2 ug/ml MWCNT for 1, 6 or 24 h. HMVEC were lysed in Lysis Buffer according to the manufacturer’s protocol (Cell Signaling Technology). Lysates were incubated with pre-coated antibodies to NF-κB, phospho-SAPK/JNK (Thr183/Tyr185), phospho-NF-κB p65 (Ser536), phospho-p38 MAPK (Thr180/Tyr182), phospho-IκB-α (Ser32), and phospho-Stat3 (Tyr705) and detected using antibodies provided by the manufacturer. Absorbance was read at 450 nm. Statistical analsysis was determined using a two-sample *t*-test assuming unequal variance.

## Abbreviations

MWCNT: Multi-walled carbon nanotubes; ROS: Reactive oxygen species; SAEC: Small airways epithelial cells; HMVEC: Human microvascular endothelial cells; DHE: Dihydroethidium; VEGFA: Vascular endothelial growth factor A; sICAM1: Soluble intracellular adhesion molecule 1; sVCAM1: Soluble vascular cell adhesion molecular 1; DM: Dispersion medium.

## Competing interests

The authors declare that they have no competing interests.

## Authors’ contributions

BNT carried out all co-culture experiments, including ROS, confocal, angiogenesis, and ELISA, participated in the study design, and drafted the manuscript. DSB performed transmission electron microscopy on co-culture samples. VC participated in the study design and helped to draft the manuscript. YQ conceived of the study, participated in the study design, and helped to draft the manuscript. NLG assisted with the study design. All authors read and approved the final manuscript.
